# Synergistic antifungal activity of minocycline as an effective augmenting agent of fluconazole against drug-resistant *Candida tropicalis*

**DOI:** 10.1128/spectrum.03185-24

**Published:** 2025-03-31

**Authors:** Yun-Zhu Zhu, Xiang Li, Qing-Yue Zhang, Ning Yang, Ping Tian, Ding Zhang, Yi Yang, Liang Yu, Yan-Yan Liu, Ying Ye, Ya-Sheng Li, Jia-Bin Li

**Affiliations:** 1 Department of Infectious Diseases & Anhui Province Key Laboratory of Infectious Diseases, The First Affiliated Hospital of Anhui Medical University 36639, Hefei, China; 2 Anhui Center for Surveillance of Bacterial Resistance & Institute of Bacterial Resistance Anhui Medical University 12485, Hefei, China; 3 Department of Gastroenterology, Linyi People’s Hospital, Linyi, Shandong, China; 4 School of Biological Sciences, The University of Hong Kong, Hong Kong SAR, China; Universita degli Studi di Modena e Reggio Emilia, Modena, Italy

**Keywords:** *Candida tropicalis*, fluconazole resistance, minocycline, synergistic effect, antifungal therapy, RNA sequencing

## Abstract

Invasive candidiasis has emerged as a significant healthcare challenge, with a rising incidence rate attributed to the widespread use of organ transplantation, chemotherapy, immunosuppressants, and broad-spectrum antibiotics. The increasing prevalence of drug-resistant strains, particularly among *Candida tropicalis*, has necessitated the exploration of novel therapeutic strategies. Our study investigated the synergistic effects of minocycline (MIN) combined with fluconazole (FLC) against FLC-resistant *C. tropicalis*, both *in vitro* and *in vivo*. The *in vitro* synergistic activity of MIN and FLC was evaluated using checkerboard titration and time-kill assays. The *Galleria mellonella* larvae and mouse model were employed to assess *in vivo* efficacy, with histopathological examination and fungal burden quantification. Whole-genome and RNA sequencing elucidated the synergistic mechanisms observed. The FLC/MIN combination significantly lowered the minimum inhibitory concentration (MIC) and improved fungicidal activity, as evidenced by enhanced survival rates and reduced fungal burden in *G. mellonella* larvae and mouse models. Histopathological analysis confirmed less tissue damage and fungal load with combination therapy. RNA sequencing analysis suggested that the impact of MIN on amino acid metabolism contributes to the synergistic effects. This approach holds promise for treating FLC-resistant *C. tropicalis* by increasing antifungal efficacy and reducing drug resistance risks, warranting further clinical exploration.

**IMPORTANCE:**

This study highlights the potential of minocycline and fluconazole combination therapy in combating drug-resistant *Candida tropicalis*. It shows promising *in vitro* and *in vivo* synergistic effects, reducing MIC and enhancing fungicidal activity. Further clinical trials are needed to validate its efficacy in treating FLC-resistant *C. tropicalis* infections.

## INTRODUCTION

In recent years, the incidence of invasive candidiasis has shown a significant increase due to increasing risk factors such as organ transplantation, cancer chemotherapy, immunosuppressive agents, and the widespread application of broad-spectrum antibiotics (1). These infections pose a serious threat to patients' lives and impose a substantial burden on the efficient utilization of medical resources (2). *Candida albicans* has been predominantly identified as the causative agent in candida infections in previous studies (3). However, recent studies have found that the epidemiological characteristics of candidiasis have undergone significant changes, and the proportion of non-*Candida albicans* infections has gradually increased (4). Pfaller et al. monitoring Candida species from 1997 to 2016 indicates that the isolation rates of *Candida tropicalis* in the Asia-Pacific region and Latin America were 14.1% and 17.0%, respectively, significantly higher than those in North America (8.0%) and Europe (7.5%). The isolation rate of *C. tropicalis* is significantly higher in Asian and Latin American regions (5). Among non-albicans candidemia cases, *C. tropicalis* candidemia exhibits the highest levels of C-reactive protein and Acute Physiology and Chronic Health Evaluation II (APACHE II) scores. Furthermore, *C. tropicalis* infection is significantly associated with a 30-day mortality rate among patients. Candidemia due to *C. tropicalis* manifests as the most severe clinical presentation and carries the worst prognosis among non-albicans candidemia (6).

Currently, four classes of antifungal agents are used in the clinical treatment of invasive candidiasis: azoles (e.g., fluconazole [FLC], voriconazole, and itraconazole), echinocandins (e.g., caspofungin), polyenes (e.g., amphotericin B), and nucleoside analogs (e.g., 5-fluorocytosine) (7). With the shortage of existing pharmaceuticals, the rise of pathogen resistance poses a substantial risk to patient health (8). FLC, the most commonly prescribed antifungal agent, is seeing its efficacy increasingly compromised by the emergence of drug-resistant strains (9). A retrospective study spanning nine years revealed that the increase in the resistance rate of *C. tropicalis* to FLC from 5.7% to 31.8% has emerged as a non-negligible issue in China (10). Furthermore, the emergence of multi-drug resistance may pose further threats. Consequently, there is an urgent need to develop new antifungal strategies to combat FLC-resistant *C. tropicalis*.

The development of novel drugs holds significant potential for breakthroughs in the current severe trend of drug resistance, yet it is characterized by long research cycles and high development risks. Consequently, the reuse of drugs already approved for other diseases has emerged as a potent approach (11). The repurposing of existing drugs, which encompasses the identification of novel applications for these drugs in the fight against antibiotic-resistant pathogens in humans, offers a cost-effective solution (7). For instance, ribavirin has been identified to possess antifungal activity against *Candida* spp. *in vitro* and could potentially serve as an adjunctive agent in antifungal therapy (12). Meanwhile, riboflavin has been demonstrated to inhibit the growth of *Candida* through multiple mechanisms and has shown efficacy in treating oropharyngeal candidiasis in animal models (13). These studies not only expand the scope of drug applications but also provide new targets and strategies for the development of novel antifungal agents, highlighting the significance of interdisciplinary collaboration in drug development. Hughes et al. have demonstrated the antifungal effects of tetracycline antibiotics, such as tetracycline, doxycycline, and minocycline (MIN), on *Aspergillus* spp. (14). Kurakado et al. demonstrated that MIN could inhibit *C. albicans* budded-to-hyphal-form transition and biofilm formation (15). In our previous research, the combination of MIN and FLC plays a synergistic role in FLC-resistant *Cryptococcus neoformans in vitro*, with the values of fractional inhibitory concentration index (FICI) ranging from 0.05 to 0.38. It can also enhance the survival rate of *Galleria mellonella* larvae post-infection *in vivo* (16). Moreover, MIN has also been shown to act synergistically with FLC when treating clinical *C. albicans* isolates both *in vitro* and *in vivo* (17). However, the synergistic effect of MIN and FLC on FLC-resistant *C. tropicalis* has not been adequately investigated.

In this paper, we explored the *in vitro* and *in vivo* activity of MIN alone and in combination with FLC against FLC-resistant *C. tropicalis*. Furthermore, RNA sequencing suggested that MIN downregulated amino acid metabolisms. The results provide valuable insights into potential synergistic mechanisms, laying a solid foundation for the development of innovative therapeutic strategies to combat resistant infections.

## MATERIALS AND METHODS

### Fungal culture conditions, strains, and reagents

FLC-resistant *C. tropicalis* (CT27) was isolated from clinical patients. The strain was preserved in a medium containing 25% glycerol at −80°C. The strain was incubated three times on Sabouraud dextrose agar (SDA) at 30°C for at least 24 h before experiments. FLC and MIN with purity of >99% were purchased from MedChemExpress. FLC was dissolved in dimethyl sulfoxide (DMSO), and the final concentration of DMSO was lower than 0.1%. MIN was soluble in water. Voriconazole (VOR), itraconazole (ITR), posaconazole (POS), caspofungin (CAS), flucytosine (5-FC), and amphotericin (AMB) were purchased from MedChemExpress. In addition, MOPS [3-(*N-morpholino*) propanesulfonic acid] and RPMI 1640 were obtained from Sigma-Aldrich.

### Synergy assay *in vitro*

#### Antifungal susceptibility test

The minimal inhibitory concentrations (MICs) of antifungal agents against *C. tropicalis* using the broth microdilution method according to Clinical and Laboratory Standards Institute M27-Ed4 (CLSI M27-Ed4, https://clsi.org/standards/products/microbiology/documents/m27/). The samples were serially twofold diluted with RPMI 1640 medium in 96-well microplates and obtained a final concentration ranging from 0.125 to 128 µg/mL. Each microwell received a 0.1 mL yeast at a concentration of 1 × 10^3^ CFU/mL in RPMI 1640 medium (pH 7.0 ± 0.1) buffered with MOPS. RPMI 1640 medium was used as a blank control, and a drug-free well was set as the growth control. The plates were then incubated at 35℃ for 24 hours. The MICs were determined as the lowest concentration of antifungal agents that resulted in at least 90% and 50% inhibition of growth compared to the control without drugs. All MIC assays were performed in duplication and repeated in three independent experiments.

### Detecting synergistic antifungal activities by the checkerboard method

The combined effect of FLC and MIN was assessed on planktonic cells using a checkerboard titration method. The experiments were carried out on 96-well round-bottom polystyrene microtiter plates with RPMI 1640 medium. The fungal concentration was diluted to 1 × 10^3^ CFU/mL in the medium, and each well contained a final volume of 0.2 mL. The concentration of FLC ranged from 1 to 128 µg/mL, while the concentration of MIN ranged from 1 to 32 µg/mL. Plates were incubated for 24 h at 35°C, and the MIC was determined as described above.

The combined effect of the drug interactions was analyzed using the FICI. The FICI was calculated using the formula FICI = (Ac/Aa) + (Bc/Ba), where Ac and Bc are the MICs of the antifungal drugs A and B in combination and Aa and Ba are the MICs of antifungal drugs A and B alone. FICI <0.5 indicates synergy, an FICI ≥0.5 and ≤4 indicates indifference, and an FICI >4 indicates antagonism. The FICI assays were carried out in triplicate.

### Time-kill assays

Fresh *C. tropicalis* colonies from an overnight growth were added to sterile phosphate-buffered saline (PBS), and the concentration of yeast was adjusted to 1 × 10^5^ CFU/mL. The yeasts were treated with FLC (16 µg/mL), MIN (2, 4, and 8 µg/mL), and combinations of FLC and MIN. The yeast treated without drug was the growth control. Each 5 mL culture (RPMI 1640 medium buffered with MOPS) was incubated at 35°C with constant shaking. A total of 100 µL samples was collected at 0, 4, 8, 12, and 24 h and then diluted 10- to 10^6^-fold with sterile PBS. The diluted samples (100 µL) were aseptically placed on SDA plates incubated at 30°C for 24 h to measure the viable CFU/mL. Each experiment was performed in triplicate.

### Quantitative detection of biofilm by crystal violet assay

The effects on biofilm formation of FLC or MIN alone and in combination were determined by reduction assay of 2,3-Bis-(2-methoxy-4-nitro-5-sulfophenyl)-2H-tetrazolium-5-carboxanilide (XTT). CT27 was inoculated in RPMI 1640 medium at 30°C overnight. The culture was centrifuged at 7,800 rpm for 5 min at 4°C and washed three times with sterile PBS. After being resuspended in RPMI 1640 medium, the yeast concentration was adjusted to 1 × 10^5^ CFU/mL. The yeast suspension was treated with FLC (1 µg/mL), MIN (1 µg/mL), or both. A sterile 24-well with fetal bovine serum incubation at room temperature for one overnight in advance. After the serum was discarded and each well was with sterile PBS, the inoculum was added to the 24-well plate and cultured in a 37°C incubator with constant shaking at 90 rpm for 4 h. The plate was then placed in the 37°C incubator without shaking for 20 h. The effect of drugs on the growth of fungal biofilm was observed. The plate was washed three times with sterile PBS to remove planktonic cells. A reduction assay of XTT was used to evaluate the effect of biofilm formation. A total of 200 µL of XTT solution (1 mg/mL in PBS) and 4 µL of a solution of menadione were added to each well. The microplate was incubated at 37°C for 2 h and measured at 492 nm (OD_492_). Each experiment was performed in triplicate.

### Efficacy assay *in vivo*

#### *G. mellonella* model

To investigate the synergistic effects of FLC and MIIN against resistant *C. tropicalis* (CT27) *in vivo*, we established an invertebrate model. Larvae with a body length of 1.5–2.5 cm, a body weight of 200–250 mg, similar and uniform color, no black spots, and vigorous vigor were selected as experimental subjects. *C. tropicalis* (CT27) inoculums were prepared from cultures grown overnight at 30°C in RPMI 1640 medium and washed three times with sterile PBS. A cell counter was utilized to calibrate the bacterial solution’s concentration to 1 × 10^8^ cells/mL in sterile PBS. Each larva was first injected with 1 × 10^7^ cells via the last left pro-leg using a 25 µL glass microsyringe. A second 10 µL injection with either FLC (160 µg/mL), MIN (50 µg/mL), FLC (160 µg/mL) + MIN (50 µg/mL), or sterile vehicle (PBS buffer) was done via the last right pro-leg 2 h post*-C. tropicalis* infection. The treatment was administered a single time. The larvae were monitored for survival at 24-h intervals over a period of 5 days. Larvae were deemed dead if they exhibited no response to tactile stimulation. Throughout the experiment, the larvae were kept in darkness at a constant temperature of 37°C.

To evaluate the effect of the fungal load on the infected larvae, the larvae were sacrificed 24 h post-treatment. They were disinfected using alcohol and homogenized in 1 mL of sterile PBS. The homogenates were subsequently diluted in a 10-fold series, after which the resulting dilutions were plated on SDA plates. Colony counts were carried out after a 24-h incubation at 30°C.

For the histopathological examination, at 24 h following treatment administration, a subset of two *G. mellonella* larvae were randomly selected from each experimental group. These larvae were fixed in formalin, subsequently embedded in paraffin, and sectioned at the midsection of their bodies to obtain representative tissue samples. The prepared sections underwent periodic acid-Schiff (PAS) staining to facilitate the visualization of inflammatory nodules within the tissue architecture.

#### Mouse model

The infection mouse model of systemic candidiasis was established as described previously with minor modifications (18). Seven-week-old specific-pathogen-free female CD-1 mice weighing 27–32 g were randomly selected and assigned to different experimental groups. The animal study was reviewed and approved by the Animal Experimentation Ethics Committee of Anhui Medical University (approval no. LLSC20190253), and the experiments were carried out in strict accordance with the Animal Research: Reporting of *In Vivo* Experiments (ARRIVE) guidelines for the care and use of laboratory animals. The experimental procedure in mice is shown in Fig. 3A. Mice were immunosuppressed by injection with cyclophosphamide (MedChemExpress) subcutaneously 4 days (150 mg/kg) and 1 day (100 mg/kg) before infection. These mice were infected with 1 × 10^6^ cells of CT27, suspended in 0.1 mL PBS via the tail vein. Subsequently, the mice were divided into four groups (*n* = 5), and 2 h after infection with CT27, they were administered FLC (7 mg/kg), MIN (5 mg/kg), FLC (7 mg/kg) + MIN (5 mg/kg), and the vehicle (sterile PBS) by intraperitoneal injection. Twenty-four hours after infection, the mice were sacrificed, and the lungs, spleens, and kidneys were removed and homogenized in 1 mL of PBS. The homogenate was serially diluted, and aliquots were plated on SDA for viable fungal colony counts after incubation for 24 h at 30°C.

### Whole-genome sequencing analysis

After activation, CT27 was grown on SDA incubated at 30°C for 24 h. After confirming the purity of strains and samples, they were used in experiments. Liquid cultures (20 mL) were cultivated in a 200 mL flask at 30°C for 24 h with shaking at 200 rpm. DNA extraction, DNA quality testing, DNA library construction and quality testing, and Nanopore sequencing were performed by Guangdong Magigene Biotechnology Co. Ltd. Sequencing data were mapped to the reference genome *C. tropicalis* MYA-3404 (https://www.ncbi.nlm.nih.gov/assembly/GCA_017315405.1). Using Flye (v2.9.2-b1786), we assembled the sequencing data into genomic sequences. Gene predictions were made with Augustus (v2.7). Annotations were performed by aligning sequences to the Gene Ontology database with HMMER (v3.1b1) and to the KOG database with diamond (v2.1.8.162). Protein sequences from the sample were clustered into representative gene families at 95% identity using CD-HIT (v4.5.8).

### RNA sample preparation and RNA sequencing (RNA-seq) analysis

*C. tropicalis* (CT27) was inoculated in an SDA plate and grew for 24 h. Single colonies were picked in YPD liquid medium and incubated overnight at 30°C with shaking. A total of 50 µL of the bacterial solution was resuspended in fresh RPMI 1640 medium, and drugs were added so that the final concentrations of each group were FLC (1 µg/mL), MIN (1 µg/mL), and FLC (1 µg/mL) + MIN (1 µg/mL). A culture with the addition of DMSO was used as a control. The culture was grown at 30°C for 10 h to the exponential growth phase. The cells were pelleted by centrifugation at 1,200 × *g* for 10 min and washed three times with sterile PBS. Afterward, the cell pellet was flash-frozen in liquid nitrogen. Triplicates were prepared for each sample. RNA extraction, rRNA depletion, cDNA library construction, and paired-end sequencing on the Illumina HiSeq 2000 platform were performed by Guangdong Magigene Biotechnology Co. Ltd. Differentially expressed genes (DEGs) were identified using the edgeR software. A fold change threshold of 2 and a false-discovery rate (FDR) of 0.05, adjusted using the Benjamini-Hochberg (BH) method, were set to filter significant DEGs.

### Quantitative real-time PCR (qRT-PCR) assay

Yeast was treated as described above. Total RNA was isolated from the cells using a YeastRNA Kit (R6870-01, Omega Bio-Tek, USA) following the manufacturer’s instructions. The total RNA was reverse-transcribed to synthesize cDNA using a PrimeScript cDNA synthesis kit (TaKaRa, Kyoto, Japan) according to the manufacturer’s recommendations. The cDNA was used in real-time qPCR with PrimeScript RT master mix (TaKaRa) on a three-step real-time PCR system (LightCycler 96, Roche, Basel, Switzerland). *ACT1* was used as an internal control gene. The primers for qRT-PCR are listed in Table S2. The changes in the fluorescence of SYBR Green and the cycle threshold (CT) were determined. The expression levels of the target genes of interest were determined by the 2^−ΔΔ*CT*^ calculation method and reported as fold change values. Experiments were run in triplicate.

### Statistical analysis

All experiments were performed three times on different dates. Statistical analyses and the generation of graphical representations were performed using GraphPad 8.0 version. The data were analyzed statistically using one-way analysis of variance, followed by Tukey’s test for comparisons involving more than two groups, or Student’s *t*-test for comparing values between two specific groups. The significance levels were set at a *P* value <0.05 (*P < 0.05, **P < 0.01, ***P < 0.001), while *P* > 0.05 was considered statistically non-significant.

## RESULTS

### Clinical *C. tropicalis* (CT27) antifungal susceptibility testing

CT27 was a strain isolated from the blood of patients with candidemia. The MICs of antifungal drugs were assessed, and the resulting data are shown in Table 1. The result suggested that CT27 was FLC-resistant, with an MIC of >128 µg/mL.

**TABLE 1:** MIC values of antifungal drugs against CT27

Antifungal drugs^*a*^	MIC_90_ (μg/mL)^*b*^	MIC_50_ (μg/mL)^*c*^
FLC	>128	128
VOR	0.25	0.125
ITR	4	2
POS	1	0.5
CAS	0.25	0.125
AMB	2	2
5-FC	<0.125	<0.125

^*a*^ FLC, Fluconazole; VOR, Voriconazole; ITR, Itraconazole; POS, Posaconazole; CAS, Caspofungin; AMB, Amphotericin B;5-FC, Flucytosine.

^*b*^ MIC_90_ refers to the minimum inhibitory concentration of a drug that inhibits fungal growth by 90% relative to the control group's growth.

^*c*^ MIC_50_ refers to the minimum inhibitory concentration of a drug that inhibits fungal growth by 50% relative to the control group's growth.

### Synergistic effects of FLC and MIN in FLC-resistant *C. tropicalis*

**Fig 1:**
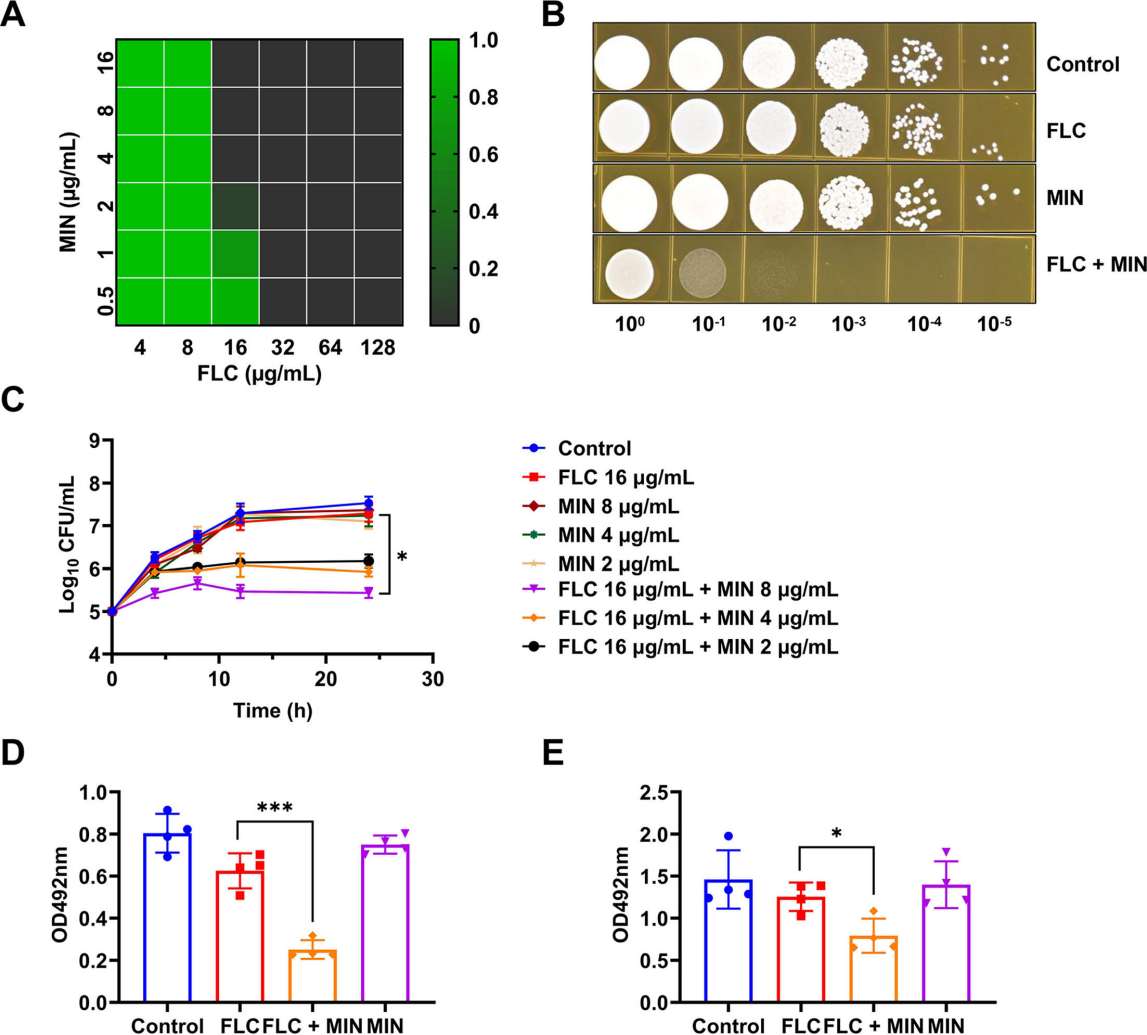
Synergistic effects of FLC and MIN in FLC-resistant *C. tropicalis*. (A) CT27 checkerboard method. (B) Serial dilution spot plate assay. CT27 was serially diluted by a factor of 10 and spotted onto SDA plates containing varying drug concentrations, incubated at 30°C for 24 h, and photographed. Control (no drug), FLC (16 µg/mL), MIN (2 µg/mL), and FLC (16 µg/mL) + MIN (2 µg/mL). (C) Time-kill curve. CT27 was cultured in RPMI 1640 liquid medium with the addition of different concentrations of FLC and MIN. Fungal viability was determined by serial dilution and cultivation on SDA solid medium at 0, 4, 8, 12, and 24 h. *n* = 3 biological replicates. (D) Biofilm growth inhibition assay. CT27 biofilm growth was monitored for 24 h in the absence of drugs, with FLC (1 µg/mL), MIN (1 µg/mL), and a combination of both. The biomass of the biofilm was quantified using the XTT reduction method by measuring absorbance at 492 nm. *n* = 4 biological replicates. (E) Biofilm eradication assay. CT27 was examined for biofilm growth after 24 h in the absence of drugs, with FLC (1 µg/mL), MIN (1 µg/mL), and a combination of both, using the XTT reduction method to quantify biofilm biomass by measuring the absorbance at 492 nm. *n* = 4 biological replicates. All data are presented as mean ± standard deviation. Error bars represent standard errors of the means. Statistically significant differences are indicated as follows: ns, no significance, *P < 0.05, **P < 0.01, and ***P < 0.001. FLC, fluconazole; MIN, minocycline; SDA, Sabouraud dextrose agar; XTT, 2,3-Bis-(2-methoxy-4-nitro-5-sulfophenyl)-2H-tetrazolium-5-carboxanilide.

The antifungal activity of MIN alone and in combination with FLC was assessed via checkerboard microdilution assays. The MIC of MIN alone was >128 µg/mL. In combination with two drugs, the presence of MIN (2 µg/mL) and FLC resulted in an eightfold reduction in the MIC, with a FICI value of 0.141. The combination of FLC and MIN had potent synergistic activity against FLC-resistant CT27 (Fig. 1A and B). To ascertain if the combination of MIN and FLC enhances fungicidal efficacy, time-kill assays were performed on the strain CT27 (Fig. 1C). The combination treatment, consisting of FLC (16 µg/mL) and MIN (8 µg/mL), demonstrated rapid and sustained fungicidal effects throughout the duration of the assay (*P* = 0.0364). The fungicidal effect of the MIN/FLU combination becomes more pronounced with increasing concentrations of MIN, demonstrating the concentration dependence of MIN. The experiment indicated that when the concentration of FLC was 1 µg/mL and the concentration of MIN was also 1 µg/mL, neither drug alone nor their combination affected the growth of CT27 (Fig S1). We used the concentrations to test the effect of MIN on biofilm formation and clearance *in vitro* to estimate its potential as an antifungal. The *in vitro* synergism of MIN in combination with FLC against biofilm formation and clearance was examined with XTT reduction assays. Compared to the effects of FLC alone, the combination of MIN and FLC exhibited a strong inhibitory effect on biofilm formation and clearance (*P* = 0.0002 and 0.0127) (Fig. 1D and E).

### Synergistic effects of FLC and MIN in *G. mellonella* larvae *Candida* infection model

**Fig 2:**
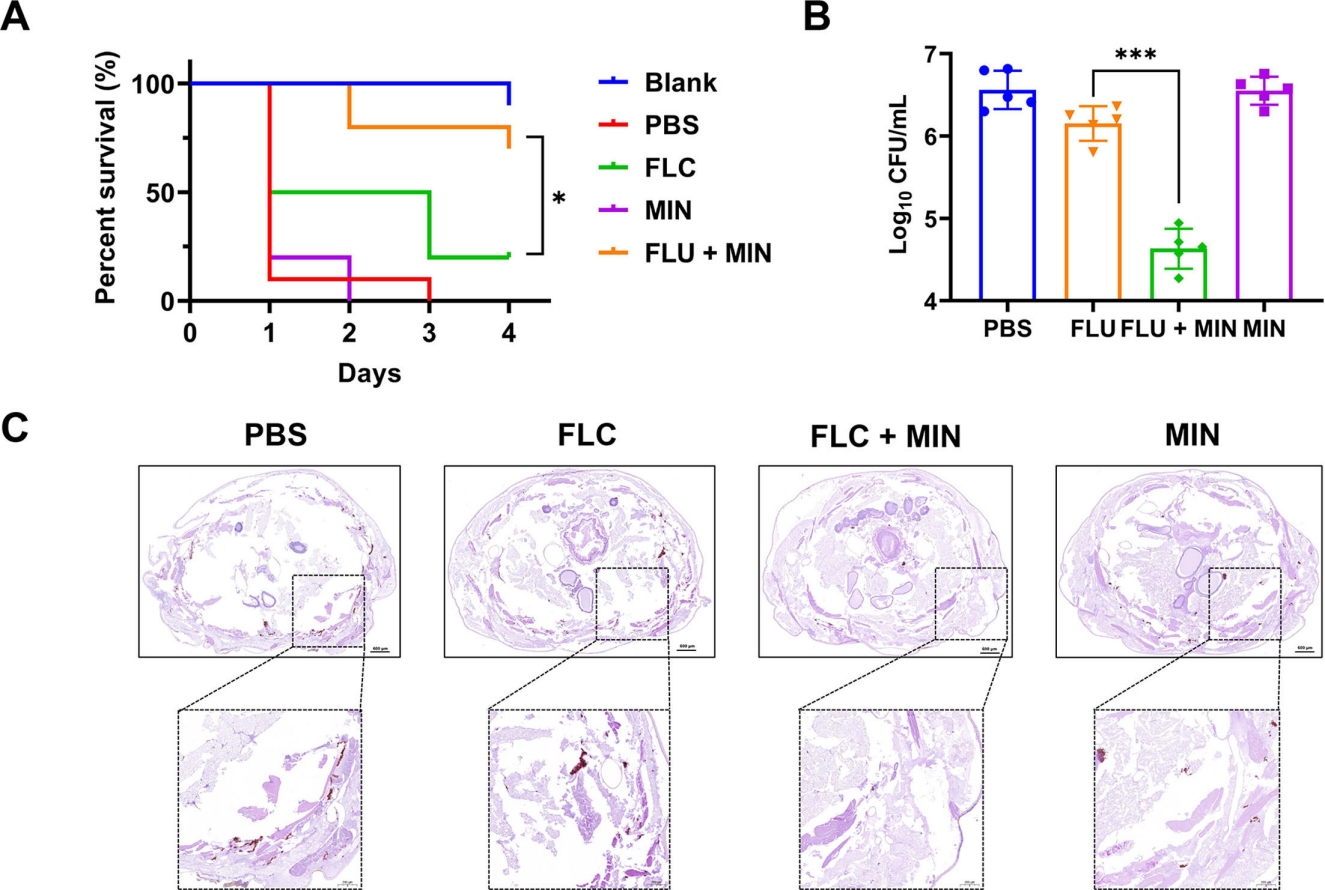
Killing effect of MIN against FLC-resistant *C. tropicalis* infection in *G. mellonella* model. (A) Survival curves of *G. mellonella* larvae infected with CT27 treated with different drugs. (B) Fungal burden in CT27-infected G. mellonella larvae after treatment with different drugs. (C) Histopathology of *G. mellonella* larvae after CT27 infection treated with different drugs. Each larva was injected with 1 × 10^6^ CT27, and 1.6 µg FLC (FLC group), 0.5 µg MIN (MIN group), and 1.6 µg FLC + 0.5 µg MIN (FLC + MIN group) were administered 2 h post-infection, with PBS group as the control. Treatment was administered once. At 24 h post-infection, five larvae from each group were randomly selected, homogenized, diluted to an appropriate concentration, and plated onto SDA plates to determine the CT27 load *in vivo*. After 24 h of infection, three larvae from each group were selected, sliced into 5 µm thick sections, stained with PAS, and observed under a microscope, with dark staining indicating inflammatory nodules. All data are presented as mean ± standard deviation. Error bars represent standard errors of the means. Statistically significant differences are indicated as follows: ns, no significance, *P < 0.05, **P < 0.01, and ***P < 0.001. PAS, periodic acid-Schiff; SDA, Sabouraud dextrose agar.

In the study, *G. mellonella* larvae infected by CT27 were used to evaluate the *in vivo* interactions of FLC/MIN combination. Compared to the model group, the groups treated with FLC or MIN monotherapy did not exhibit significant changes in survival rates and fungal burden. On day 4, the survival rates of the combination therapy group were significantly higher than those of the other three groups (*P* = 0.0177) (Fig. 2A). And the FLC/MIN treatment group had significantly reduced colony counts in 24 h after infection (*P* < 0.0001) (Fig. 2B). Histopathological examination of *G. mellonella* larvae infected with CT27 indicated a high subcutaneous fungal load in the control group (Fig. 2C). Severe tissue destruction and large quantities of *C. tropicalis* cells were observed surrounded by hemocytes in inflammatory nodules in the PBS group. Additionally, there were numerous candida cell nodules visible in the histology sections of the drug-monotherapy groups, in contrast with the much lower amount visible in the sections from the FLC/MIN group, suggesting that monotherapy with MIN or FLC has a limited effect against FLC-resistant *C. tropicalis*. Notably, FLC/MIN could significantly reduce the number of FLC-resistant *C. tropicalis* cell nodules in infected *G. mellonella* larvae, illustrating the high efficacy of FLC/MIN combination treatments.

### Synergistic effects of FLC and MIN in mouse model of systemic candidiasis

**Fig 3:**
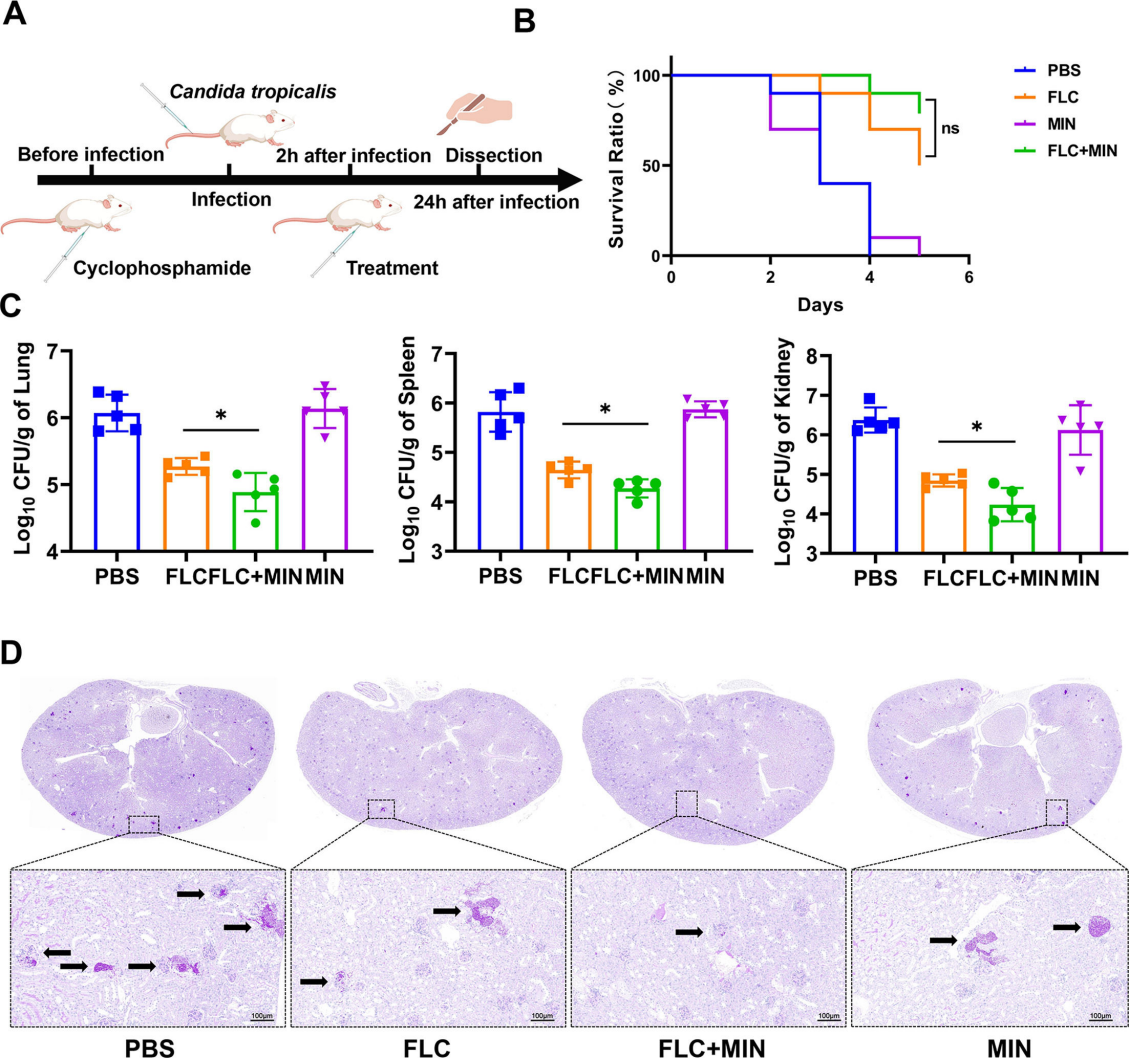
Killing effect of MIN against FLC-resistant *C. tropicalis* infection in mouse model. (A) The experimental procedure in mice. (B) Survival curves of mice infected with CT27 treated with different drugs. (C) PAS-stained kidney after CT27 infection treated with different drugs. The mice were randomly assigned to one of the four treatment groups: a control group receiving sterile phosphate-buffered saline (PBS), a group treated with FLC alone at a dosage of 7 mg/kg, a group treated with MIN alone at a dosage of 5 mg/kg, and a group treated with the combination of FLC (7 mg/kg) and MIN (5 mg/kg). All treatments were administered via intraperitoneal injection, commencing 2 h post-infection. All data are presented as mean ± standard deviation. Error bars represent standard errors of the means. Statistically significant differences are indicated as follows: ns, no significance, *P < 0.05. FLC, fluconazole; MIN, minocycline; PAS, periodic acid-Schiff.

In order to assess the therapeutic efficacy of the combination therapy with MIN and FLC in a systemic candidiasis mouse model, we established an *in vivo* model by inoculating mice with FLC-resistant *C. tropicalis*. The mice were randomly assigned to one of the four treatment groups: a control group receiving sterile PBS, a group treated with FLC alone at a dosage of 7 mg/kg, a group treated with MIN alone at a dosage of 5 mg/kg, and a group treated with the combination of FLC (7 mg/kg) and MIN (5 mg/kg). All treatments were administered via intraperitoneal injection, commencing 2 h post-infection (Fig. 3A). One day after infection, the mice were sacrificed, and the fungal burden in the lungs, spleens, and kidneys was analyzed (Fig . S2). As depicted in Fig. 3BFig. 3B, the survival rate of the FLC/MIN group was highest on the fifth day after infection (*P* = 0.4302). However, when used in combination, the FLC/MIN group resulted in a remarkable decrease in fungal burden in the lungs, spleens, and kidneys compared to mice receiving FLC only (*P* = 0.0255, 0.0103, and 0.0158). This suggests that MIN effectively restored the sensitivity of FLC against FLC-resistant *C. tropicalis in vivo*. The findings from the fungal burden analysis were corroborated by the PAS staining of kidney tissues. In the cohort subjected to the combination therapy of MIN and FLC, the typical invasion of *C. tropicalis* hyphae was notably absent (Fig. 3C).

### Genome sequencing and assembly of CT27

**Fig 4:**
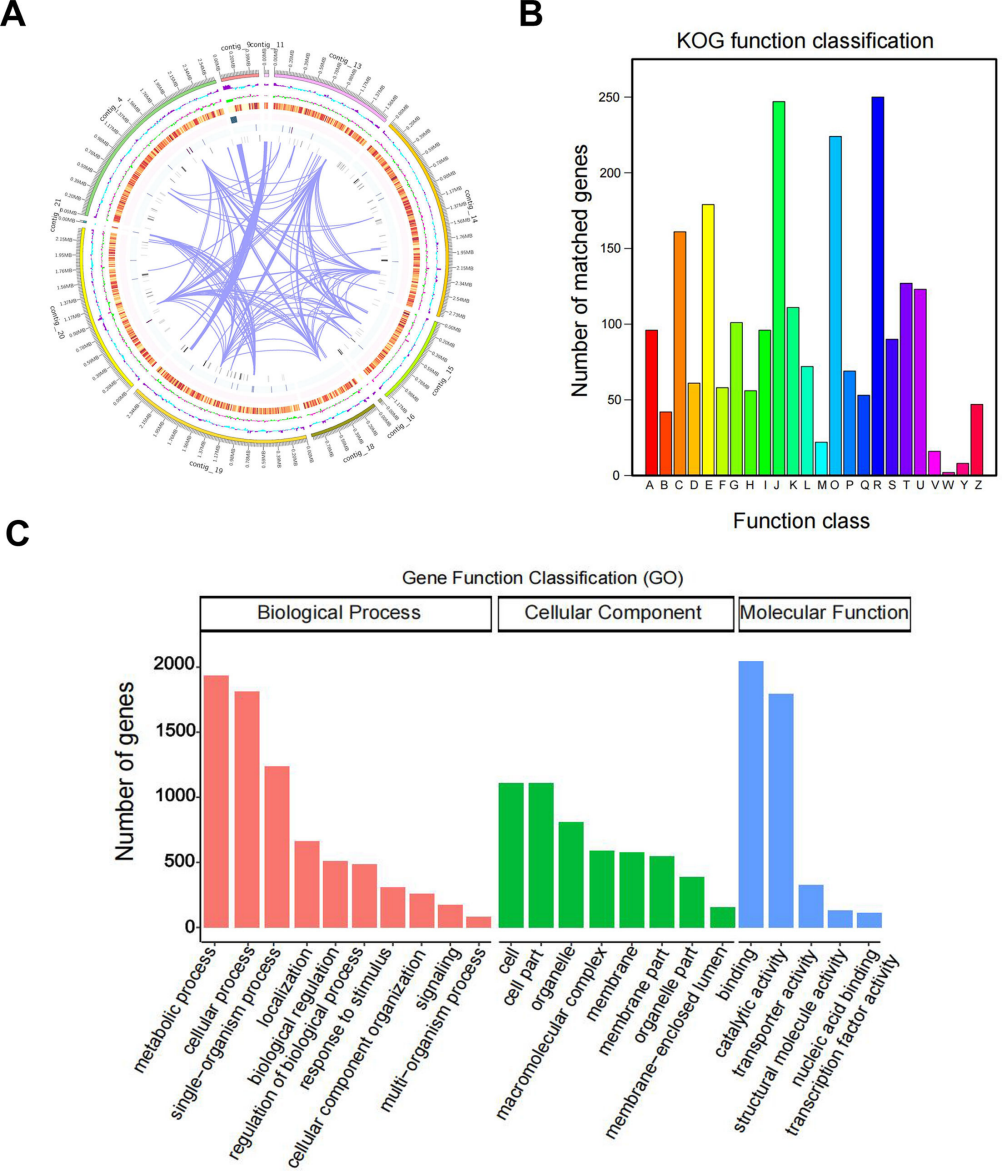
Whole-genome analysis of CT27. (A) Circos plot of the CT27 genome. The outermost ring represents the genomic sequence position coordinates, followed by GC content, GC-skew (gene density of coding genes, rRNA, snRNA, and tRNA), and gene duplication from outer to inner. (B) The annotation results from the CT27 KOG database. Functional annotations are as follows: A: RNA processing and modification (96), B: Chromatin structure and dynamics (42), C: Energy production and conversion (161), D: Cell cycle control, cell division, chromosome partitioning (61), E: Amino acid transport and metabolism (179), F: Nucleotide transport and metabolism (101), G: Carbohydrate transport and metabolism (101), H: Coenzyme transport and metabolism (56), I: Lipid transport and metabolism (96), J: Translation, ribosomal structure and biogenesis (247), K: Transcription (111), L: Replication, recombination and repair (72), M: Cell wall/membrane/envelope biogenesis (19), O: Posttranslational modification, protein turnover, chaperones (224), P: Inorganic ion transport and metabolism (69), Q: Secondary metabolite biosynthesis, transport and catabolism (53), R: General function prediction only (250), S: Function unknown (90), T: Signal transduction mechanisms (127), U: Intracellular trafficking, secretion, and vesicular transport (123), V: Defense mechanisms (16), W: Extracellular structures (2), Y: Nuclear structure (8), Z: Cytoskeleton (47). (C) The annotation results from the CT27 GO database.

To decipher the molecular characteristics of CT27, genome sequencing was performed, and the genome was assembled. Sequencing of the CT27 sample raw data using Flye (version 2.9.2-b1786) gets *C. tropicalis* genome-wide assembling. Genome assembly result statistics are as follows: the total length was 14,807,527 bp, *N*_50_ was 2,488,716 bp, *N*_90_ was 954,286 bp, and the GC content was 33.33%. Augustus (version 2.7) was used for gene prediction, rRNAmmer software (version 1.2) was used for rRNA prediction, and tRNAscan-SE software (version 1.3.1) was used for tRNA prediction. snRNA prediction was performed with Rfam software for Rfam database alignment annotation, and the final snRNA was determined using the cmsearch (version 1.1rc4) program (default parameters). The sequencing data were assembled, and 14 contigs were obtained (Table S2). A total of 11 contigs with a length of more than 20 kb were screened for genome circle mapping (Fig. 4A). A total of 2,311 genes were classified into protein functional categories based on KOG analysis. Except for class R genes of “general function prediction only” (250), the class J genes of “translation, ribosomal structure and biogenesis” had the highest number of genes (247), followed by the class O genes of “posttranslational modification, protein turnover, chaperones” (224), the class E genes, which are related to amino acid transport and metabolism (179), and the class C genes which are involved in energy production and conversion (161) (Fig. 4B). The GO functional analysis was performed to better understand the functions encoded in the CT27 (Fig. 4C). A total of 3,663 genes have been annotated with the GO database. The class with the greatest enrichment was “binding” (2,044), followed by the class of “metabolic process” (1,933), the class of “cellular process” (1,815), the class of “catalytic activity” (1,798), and the class of “single-organism process” (1,236). The sequencing data generated in this study have been deposited in the NCBI Sequence Read Archive (SRA) under accession number PRJNA1171791.

### RNA-seq analysis of potential mechanisms of synergy of FLC and MIN

**Fig 5:**
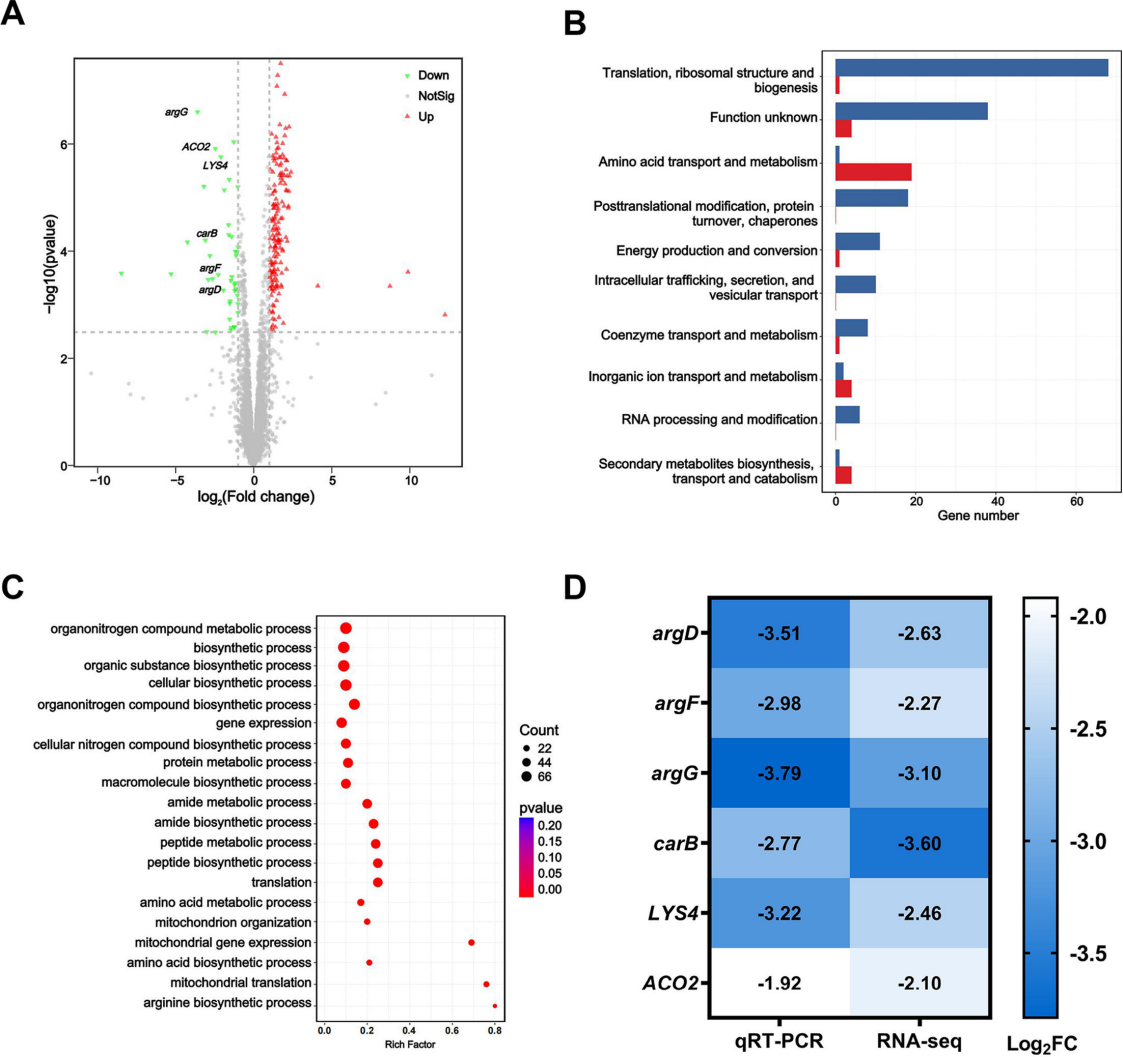
Transcriptomic alterations in CT27 induced by minocycline. (A) Volcano plot of differentially expressed genes (DEGs) between the fluconazole combined with minocycline (FCM) treatment group and the fluconazole-only (F) treatment group. Differential expression analysis was performed using the DESeq2 software, with default screening conditions of FDR ≤0.05 and |log2FC| ≥1, applying the Benjamini-Hochberg method for multiple testing corrections. (B) eggNOG annotation statistics of DEGs between the FCM and F groups. (C) GO functional enrichment analysis statistics of DEGs between the FCM and F groups. (D) Illustrates seven significantly downregulated genes. DEGs related to amino acid metabolism were selected. qRT-PCR analysis was performed on the expression of these seven selected genes. Data are presented as means of three biological replicates. FDR, false-discovery rate; qRT-PCR, quantitative real-time PCR.

To better understand the mechanism of MIN on CT27, an RNA-seq analysis was conducted. The FLC/MIN group had 173 genes upregulated and 42 genes downregulated compared to FLC alone (Fig. 5A). Differential gene expression analysis was performed using the DESeq2 software. The default screening criteria for DEGs are: FDR ≤0.05 and |log2FC| ≥1, with the method of multiple testing correction being the BH procedure. After obtaining the DEGs, they were compared to the EggNOG database to perform a functional classification analysis of the genes in the gene set. Compared to the FLC group, the class that is most downregulated in the FLC/MIN group is “Amino acid transport and metabolism,” which includes 19 genes (Fig. 5B). GO is a widely used gene function classification system. Using DEGs as the subject of interest and all expressed genes as the background, we conducted a GO functional enrichment analysis, integrating annotation information. GO functional enrichment analysis revealed significant differences in amino acid metabolism pathways between the FLC alone and FLC/MIN groups (Fig. 5C). Based on these results, we hypothesized that MIN augments the inhibition of amino acid metabolism by FLC, thereby inhibiting the growth of resistant CT27. The sequencing data generated in this study have been deposited in the NCBI SRA under accession number PRJNA1175064.

### qRT-PCR of genes related to potential mechanisms of synergy of FLC and MIN

In order to verify the results of RNA-seq analysis, seven genes that had been clearly studied in CT27 were selected for further qRT-PCR validation. There are seven differential genes related to amino acid metabolism, namely *argD*, *argG*, *carB*, *LYS4*, *argF*, and *ACO2*, which were consistent with the expression trends observed in RNA-seq (Fig. 5D).

## DISCUSSION

The currently clinically available antifungal drugs are limited in variety, have a narrow antibacterial spectrum (20). Traditional antifungal medications have the potential to induce cytotoxic effects in humans, particularly impacting the kidneys and liver (21, 22). In addition, there has been a noted rise in the occurrence of fungal resistance against major classes of antifungal agents (19). The development of novel antifungal drugs is a costly and time-consuming process, requiring significant financial and temporal investments, accompanied by uncertainties and risks. Drug repurposing offers a rapid and cost-effective alternative, significantly reducing research and development costs and risks, thus avoiding the ground-up drug discovery process compared to developing entirely new drugs (23).

MIN, a derivative of tetracycline, serves as a broad-spectrum antibacterial agent that inhibits bacterial protein synthesis (24). Due to its lipid-soluble nature, MIN can easily cross the blood-brain barrier to enter the central nervous system, exhibiting extensive antibacterial activity against both aerobic and anaerobic gram-positive and gram-negative microorganisms (25). In the 1970s, studies reported that MIN had the ability to inhibit the growth of *C. albicans*, demonstrating synergistic effects *in vitro* when combined with amphotericin B (26, 27). Further research has demonstrated the synergistic effect of MIN combined with FLC in inhibiting biofilm production (17). MIN combined with azole drugs exhibits synergistic effects both *in vitro* and *in vivo* against azole-resistant *Aspergillus fumigatus*, *Aspergillus flavus,* and *C. neoformans* (16, 28). Consistent with other studies, we found that MIN was able to enhance *C. tropicalis* sensitivity to azole treatment both *in vitro* and *in vivo*.

The checkerboard assays *in vitro* indicated that MIN exhibited a synergistic effect with FLC against FLC-resistant *C. tropicalis*. The results of time-kill experiments demonstrated that the combination of MIN and FLC significantly enhanced the antifungal ability compared to FLC alone. This synergistic effect was manifested not only in the reduction of viable bacterial counts but also in the inhibition of biofilm formation. Biofilm, a protective structure formed by *C. tropicalis* on host tissues, effectively resists antibiotic invasion (29). However, the combination of MIN and FLC significantly inhibited biofilm formation, thereby disrupting this protective structure and facilitating the entry of antibiotics into the cell interior to exert their bactericidal effect.

The *G. mellonella* larva infection model, an insect infection model, is frequently utilized in studying pathogen virulence, drug efficacy, and toxicity. Featuring both cellular and humoral defenses, similar to the innate immune response in mammals, and possessing significant economic advantages, *G. mellonella* larvae serve as an ideal infection model (30).

In this study, we employed the *G. mellonella* larvae model to evaluate the *in vivo* effects of FLC/MIN. After 4 days of infection, the larval survival rates in the MIN, FLC, and FLC/MIN groups were 0%, 20%, and 70%, respectively, indicating that FLC alone exhibited a weaker protective effect on larvae, whereas FLC/MIN significantly improved the survival rate of infected larvae. The results of fungal burden analysis and histopathological studies also indicate that the combination of MIN and FLC can enhance the protective effect against larvae infected with FLC-resistant *C. tropicalis*. Specifically, the fungal burden in the FLC/MIN group was significantly lower than that in the FLC group. Furthermore, both tissue destruction and inflammatory nodules were significantly reduced in the FLC/MIN group compared to the FLC group, suggesting less severe tissue damage in the FLC/MIN group. The consistency between *in vitro* and *in vivo* results was demonstrated: the combination of FLC and MIN enhanced the efficacy of FLC against resistant *C. tropicalis in vivo*, consistent with the findings of *in vitro* studies.

To further investigate the *in vivo* effects of MIN, this study established a mouse model infected with FLC-resistant *C. tropicalis*. Based on this model, the study validated that combination therapy with FLC/MIN could protect CD-1 mice against FLC-resistant *C. tropicalis* infection. The combined use of FLC/MIN improved mouse survival rates. The bacterial load in target organs such as the lungs, spleen, and kidneys confirmed that the combined use of MIN and FLC reduced the bacterial load in mice. FLC/MIN combination therapy provided significant protection for CD-1 mice infected with FLC-resistant *C. tropicalis*.

To further unravel the pathogenic mechanisms of the CT27 strain, our study employed high-throughput sequencing technology to perform whole-genome sequencing. This comprehensive genomic analysis is instrumental in investigating the biological characteristics, pathogenic processes, and drug resistance patterns of tropical *Candida* species. The KOG annotation results indicate a significant enrichment of gene functions in categories such as “translation, ribosomal structure and biogenesis,” “posttranslational modification, protein turnover, chaperones,” and “amino acid transport and metabolism” (Fig. 4B). The GO annotation results further reveal that the gene functions of CT27 are predominantly enriched in “metabolic process” and “cellular process” within the biological process (BP) category (Fig. 4C). Our study, therefore, underscores the importance of these pathways in the pathogenicity of CT27 and highlights the potential of targeting these processes for therapeutic interventions.

By comparing the results of RNA-seq and qRT-PCR, we observed a significant downregulation of amino acid synthesis and metabolic functions in CT27 by MIN, primarily associated with arginine and lysine (Fig. 5B through D). Genes such as *argD*, *argF*, *argG*, *and carB* are primarily linked to the arginine biosynthesis pathway (31, 32). The functions of the *LYS4* gene in yeast are primarily associated with the biosynthesis of lysine (33). Lysine, an essential amino acid, plays a crucial role in cellular growth and division. Gabriel et al.’s research demonstrates that strains with defects in the *LYS4* gene lead to deficiencies in lysine biosynthesis, thereby affecting the biofilm formation of *C. albicans* (34). Mutant strains exhibit reduced biofilm cell viability and a looser biofilm structure compared to the wild type. The mitochondrial enzyme encoded by the *ACO2* gene plays a pivotal role in the tricarboxylic acid cycle, which is crucial for cellular energy metabolism (33).

This discovery offers novel insights into the treatment of FLC-resistant *C. tropicalis*. The combined use of MIN and FLC can effectively enhance treatment efficacy and reduce the incidence of drug resistance. Moreover, this combination therapy reduces antibiotic dosage and mitigates the risk of adverse drug reactions, improving the overall treatment experience for patients. Nonetheless, the drug dosages and administration frequencies utilized in this study were derived from experimental animal models, which necessitate further optimization for clinical application. We anticipate future studies to further explore the efficacy and safety of this combination therapy, providing more options for clinical treatments.

### Conclusions

In summary, the study indicates that the combination of MIN and FLC is an effective treatment against FLC-resistant *C. tropicalis*. We consider MIN as a clinically promising agent against FLC-resistant *C. tropicalis* infections. This study serves as an early foundation for further clinical trial validation. Further analysis of dosing frequency and optimal dosage is required to achieve better clinical outcomes.

## Data Availability

The data sets used and analyzed during the current study are available from the corresponding author upon reasonable request.

## SUPPLEMENTAL MATERIAL

Supplemental material spectrum.03185-24-s0001.doc Fig. S1 and S2; Tables S1 and S2.

ASM does not own the copyrights to Supplemental Material that may be linked to, or accessed through, an article. The authors have granted ASM a non-exclusive, world-wide license to publish the Supplemental Material files. Please contact the corresponding author directly for reuse.
