# Supplementary material for: Synergistic antifungal activity of minocycline as an effective augmenting agent of fluconazole against drug-resistant Candida tropicalis
Source: Microbiol Spectr. 2025 Mar 31;13(5):e03185-24. doi: 10.1128/spectrum.03185-24 (PMC12054018; doi:10.1128/spectrum.03185-24)
Supplement: Supplemental material — Fig. S1 and S2; Tables S1 and S2. [file spectrum.03185-24-s0001.doc]

**Synergistic Antifungal Activity of Minocycline as an Effective Augmenting Agent of Fluconazole against Drug-resistant *Candida tropicalis***

**Yun-Zhu Zhu****^1,2,†^, Xiang Li^1,2,†^, Qing-Yue Zhang^1,2^, Ning Yang^1,2^, Ping Tian^3^, Ding Zhang^1,2^, Yi Yang^1,2^, Liang Yu^1,2^, Yan-Yan Liu^1,2^, Ying Ye^1,2^, Ya-Sheng Li^1,2,4^* , and Jia-Bin Li^1,2^***

^1^Department of Infectious Diseases & Anhui Center for Surveillance of Bacterial Resistance, The First Affiliated Hospital of Anhui Medical University, Hefei, 230022, China;

^2^Anhui Province Key Laboratory of Infectious Diseases & Institute of Bacterial Resistance, Anhui Medical University, Hefei, 230022, China;

^3^Department of Gastroenterology, Linyi People’s Hospital, Linyi, Shandong Province, China.

**^4^**School of Biological Sciences, The University of Hong Kong, Pokfulam Road, Hong Kong SAR, China.

*Correspondence: [lijiabin@ahmu.edu.cn](mailto:lijiabin@ahmu.edu.cn) (J.L.) & liyasheng@ahmu.edu.cn (Y.L.)

†The authors contributed equally to this study

**Table Legends**

**Table S1** Description of the genes and primer sequences

**Table S2** Description of the genes and primer sequences

**Figure Legends**

**Figure S1.** The growth curve of CT27 under various drug concentrations was analyzed. CT27 was cultured in RPMI 1640 medium with the addition of different concentrations of FLC and MIN. The fungal viability was determined through serial dilutions and cultivation on SDA solid medium at 0h, 4h, 8h, 12h, and 24h.

**Figure S2.** Fungal burden of CT27-infected mice in different organs after treatment with different drugs. The mice were randomly assigned to one of four treatment groups: a control group receiving sterile phosphate-buffered saline (PBS), a group treated with FLC alone at a dosage of 7 mg/kg, a group treated with MIN alone at a dosage of 5 mg/kg, and a group treated with the combination of FLC (7 mg/kg) and MIN (5 mg/kg). All treatments were administered via intraperitoneal injection, commencing 2 hours post-infection. One day after infection, the mice were sacrificed, and the fungal burden in the lungs, spleens and kidneys was analyzed.

**Table S1 Description of the genes and primer sequences**

| Genes | Description | Primers Sequence (F) | Primers Sequence (R) |
| --- | --- | --- | --- |
| *carB* | carbamoyl-phosphate synthase arginine-specific large chain | GACTTGTTTGCTCGTGCCTTGAAG | AGCCTCCAATGCGTCATCAACAG |
| *argG* | argininosuccinate synthase | TCTCCAGTTGACACCAGTGGTTTC | CCCTTTTCTCTGGCAGCTTCACC |
| *argD* | acetylornithine aminotransferase | TACATCAGGTATTGCCGTTACATG | TTCAGATAATTCTCCTGCTGGTTG |
| *LYS4* | homoaconitate hydratase | AGCGTACTTGGCATCACCAGAAG | TCGGCAGCAGATTCTTTGGGTTG |
| *argF* | homocitrate synthase | ACCAAGACATCAAGAAGAAGTTGC | ATCCATTGATGACGGCCATAGC |
| *ACO2* | aconitate hydratase | TGGTGGTGCTGATGCTGTTGATG | TGACGGTCAAGATACCAGCCAATG |

**Table S2 Description of the genes and primer sequences**

| contig name | Length (bp) |
| --- | --- |
| contig_1 | 10285 |
| contig_4 | 2648697 |
| contig_9 | 514063 |
| contig_10 | 17347 |
| contig_11 | 63021 |
| contig_13 | 1659823 |
| contig_14 | 2826809 |
| contig_15 | 1226686 |
| contig_16 | 46486 |
| contig_17 | 3614 |
| contig_18 | 954286 |
| contig_19 | 2488716 |
| contig_20 | 2322885 |
| contig_21 | 24809 |


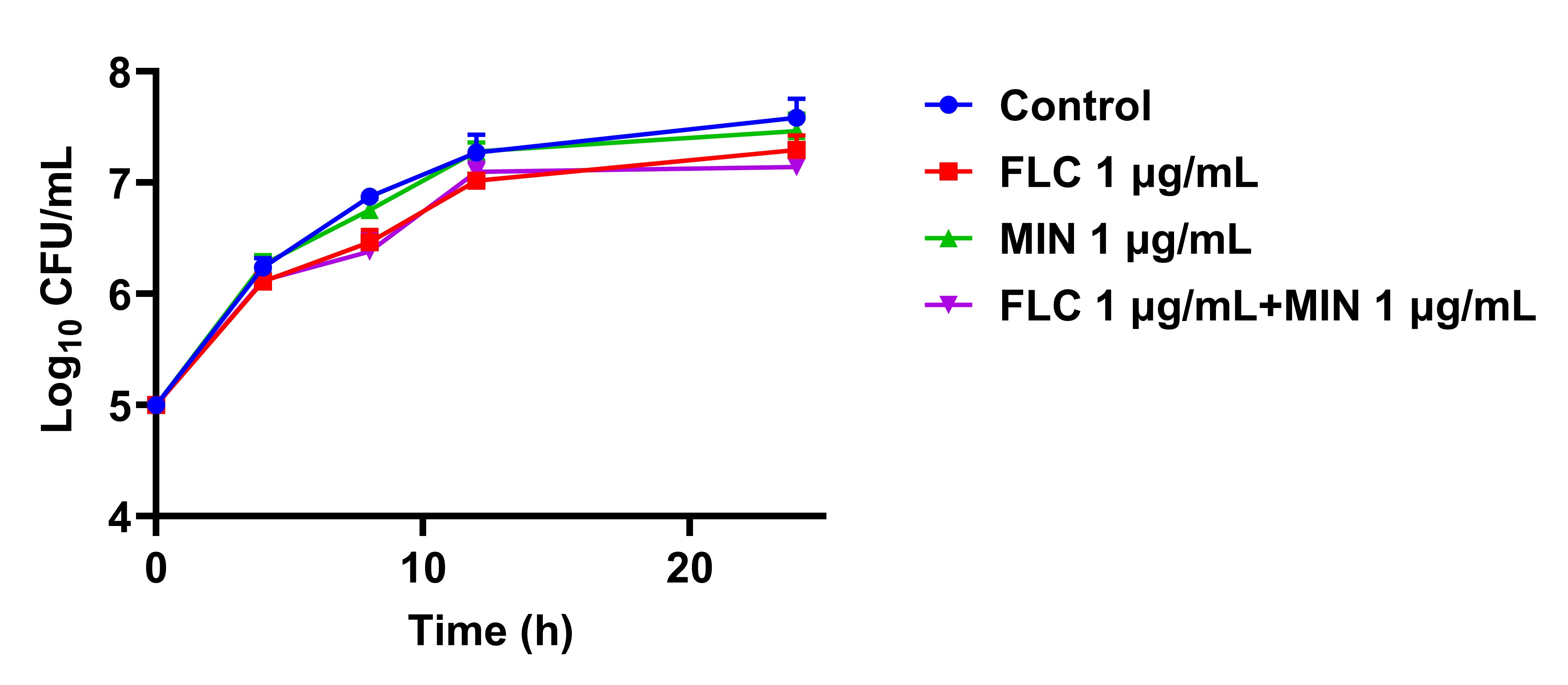


**Figure S1.** The growth curve of CT27 under various drug concentrations was analyzed. CT27 was cultured in RPMI 1640 medium with the addition of different concentrations of FLC and MIN. The fungal viability was determined through serial dilutions and cultivation on SDA solid medium at 0h, 4h, 8h, 12h, and 24h.


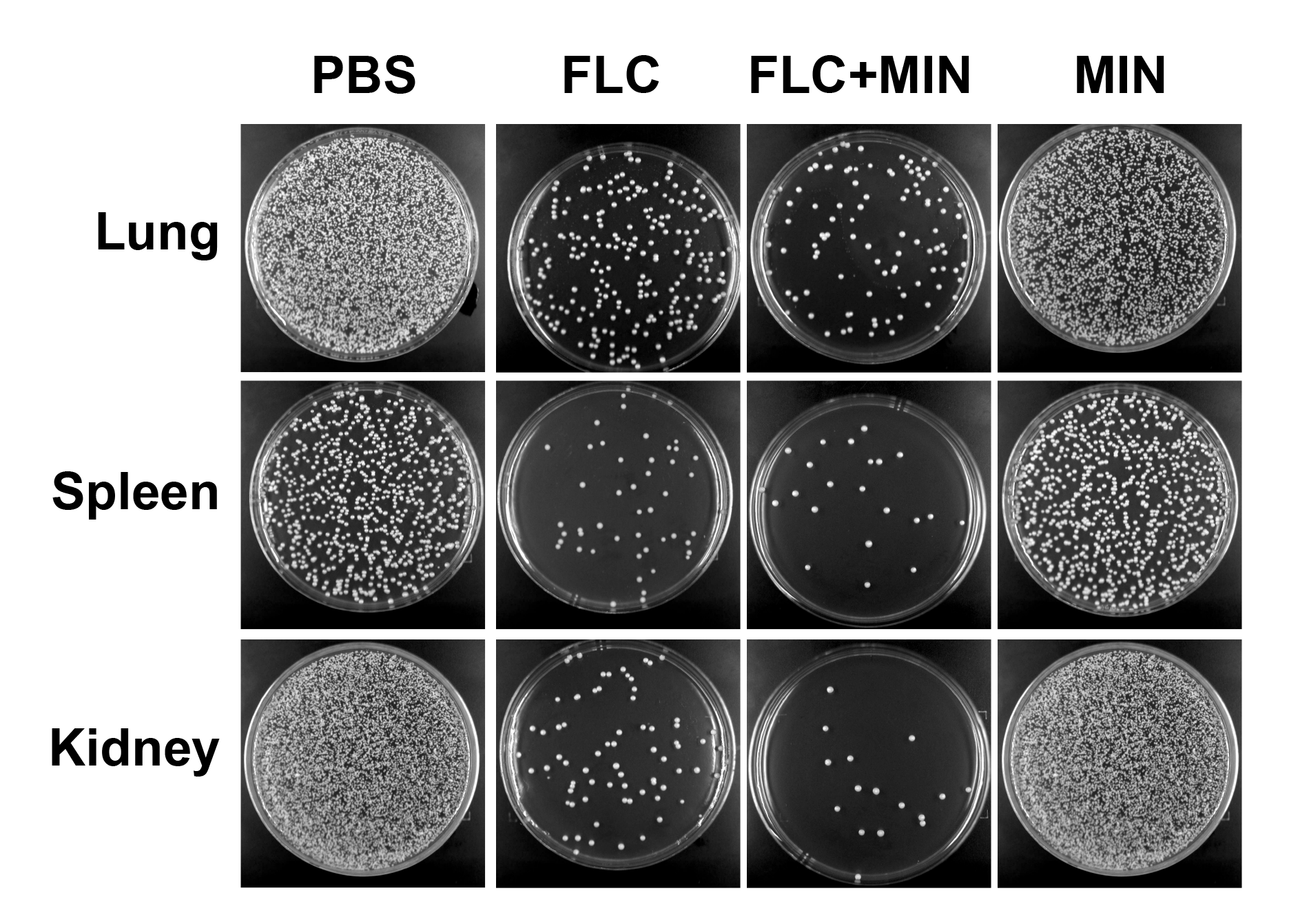


**Figure S2.** Fungal burden of CT27-infected mice in different organs after treatment with different drugs. The mice were randomly assigned to one of four treatment groups: a control group receiving sterile phosphate-buffered saline (PBS), a group treated with FLC alone at a dosage of 7 mg/kg, a group treated with MIN alone at a dosage of 5 mg/kg, and a group treated with the combination of FLC (7 mg/kg) and MIN (5 mg/kg). All treatments were administered via intraperitoneal injection, commencing 2 hours post-infection. One day after infection, the mice were sacrificed, and the fungal burden in the lungs, spleens and kidneys was analyzed.
